# MiR-21-5p and miR-223-3p as Treatment Response Biomarkers in Pediatric Eosinophilic Esophagitis

**DOI:** 10.3390/ijms26073111

**Published:** 2025-03-28

**Authors:** Antonietta Tarallo, Marianna Casertano, Anna Valanzano, Sabrina Cenni, Mara Creoli, Giuseppina Russo, Carla Damiano, Annamaria Carissimo, Alessandro Cioce, Massimo Martinelli, Erasmo Miele, Annamaria Staiano, Dario Iafusco, Giancarlo Parenti, Caterina Strisciuglio

**Affiliations:** 1 Department of Translational Medical Sciences, University of Naples “Federico II”, 80131 Naples, Italy; 2 Department of Woman, Child and General and Specialist Surgery, University of Campania “Luigi Vanvitelli”, 80138 Naples, Italy; 3 Istituto per le Applicazioni del Calcolo “Mauro Picone”, 80131 Naples, Italy; 4 Pathology Unit, Department of Mental and Physical Health and Preventive Medicine, University of Campania “Luigi Vanvitelli”, 80138 Naples, Italy

**Keywords:** eosinophilic esophagitis, microRNA, biomarkers

## Abstract

The diagnosis and monitoring of eosinophilic esophagitis (EoE), a common pediatric pathology, typically involves invasive procedures such as an upper endoscopy with biopsies, imposing a significant burden on patients and healthcare systems. We aimed to assess miR-21-5p and miR-223-3p levels in pediatric EoE patients and evaluate their as potential non-invasive biomarkers of disease activity and response to treatments. We enrolled 13 children with EoE and 8 controls. Plasma and esophageal mucosa samples from patients were collected at diagnosis and after 8–10 weeks of therapy and compared with control samples. After microRNA(miRNA) extraction, the levels of miR-21-5p and miR-223-3p and their relevant target genes were analyzed. Bioinformatic analysis was used to identify the predicted target genes and pathways that are potentially relevant for disease pathophysiology. Plasma levels of miR-21-5p and miR-223-3p were significantly higher in EoE patients than in the controls, reflecting their levels in esophageal mucosa. The target genes of these miRNAs are involved in key signaling pathways (MAPK, Ras, and FoxO), relevant for EoE pathophysiology. Among these, *STAT3 (Signal Transducer and Activator of Transcription 3)* and *PTEN (Phosphatase and Tensin Homolog)*, which are significantly downregulated in patient esophageal mucosa, are implicated in eosinophilic gastroenteropathies and autoimmune diseases. Following therapy (proton pump inhibitors and/or fluticasone propionate), plasma and tissue expression of both miRNAs significantly decreased and were no longer different from the controls. These microRNAs may serve as complementary non-invasive EoE markers and reduce the need for endoscopy/biopsies.

## 1. Introduction

Eosinophilic esophagitis (EoE) is a chronic, immune-mediated esophageal disorder characterized by eosinophilic infiltration of the esophageal mucosa and symptoms of esophageal dysfunction [1,2]. Growing awareness of and considerable interest in this disorder are emerging because of its prevalence, estimated in 0.5 to 1 cases per 1000 [3], and because of its incompletely known pathophysiology. The diagnosis and surveillance of EoE are mainly based on histological evaluations that require invasive procedures such as endoscopy with multiple biopsies, which impact substantially on patients’ quality of life. Thus, there is an urgent need for non-invasive, measurable, and reliable biomarkers that may provide support in diagnosis, in the management of patients, and in assessing their response to therapies [4]. However, biomarkers that fully meet these criteria have not yet been identified. Recently, microRNAs (miRNAs) have emerged as potential biomarkers in several fields of human disease, such as cancer, chronic inflammatory or degenerative diseases [5], and inborn genetic and metabolic defects [6]. MiRNAs are a growing class of endogenous small non-coding single- stranded RNAs that regulate gene expression by targeting messenger RNAs for translational repression or transcript degradation [7], thus modulating multiple cellular pathways. They regulate fundamental physiological and pathological processes such as cell proliferation, differentiation, apoptosis, and inflammation, including pathways potentially involved in esophageal disease. MiRNA levels are not only dysregulated in tissues but can also be measured in plasma. In recent years, it has been shown that miRNA content in circulation is likely to provide a read-out of altered pathways in response to several disease conditions [8]. Several studies have documented the dysregulation of specific miRNAs in esophageal diseases [9] and EoE, both in esophageal biopsies and in biological fluids such as blood and saliva [9,10,11,12]. In a few of these studies, miR-21-5p and miR-223-3p were shown to be upregulated in the esophageal mucosa of adult patients with EoE [13,14].

The aim of our study was to evaluate whether plasma miR-21-5p and miR-223-3p may also represent potential markers in pediatric patients, in whom invasive procedures are challenging and have more of an impact than in adults; whether their plasma levels are reflective of their tissue levels and of the esophageal pathology; and whether changes in their levels in plasma reflect the reversal of mucosal pathology in response to therapy.

## 2. Results

We studied a population of 13 subjects (Table 1) (mean age 12.5 years, 61% males, 39% females) with an established diagnosis of EoE and 8 controls (non-EoE subjects with a conclusive diagnosis of functional dyspepsia). In total, 69% of the EoE subjects also presented with atopic comorbidities such as asthma, allergic rhinitis, or atopic dermatitis. The mean maximum eosinophil count/HPF was 32.3 among the EoE subjects.

**Figure 1:**
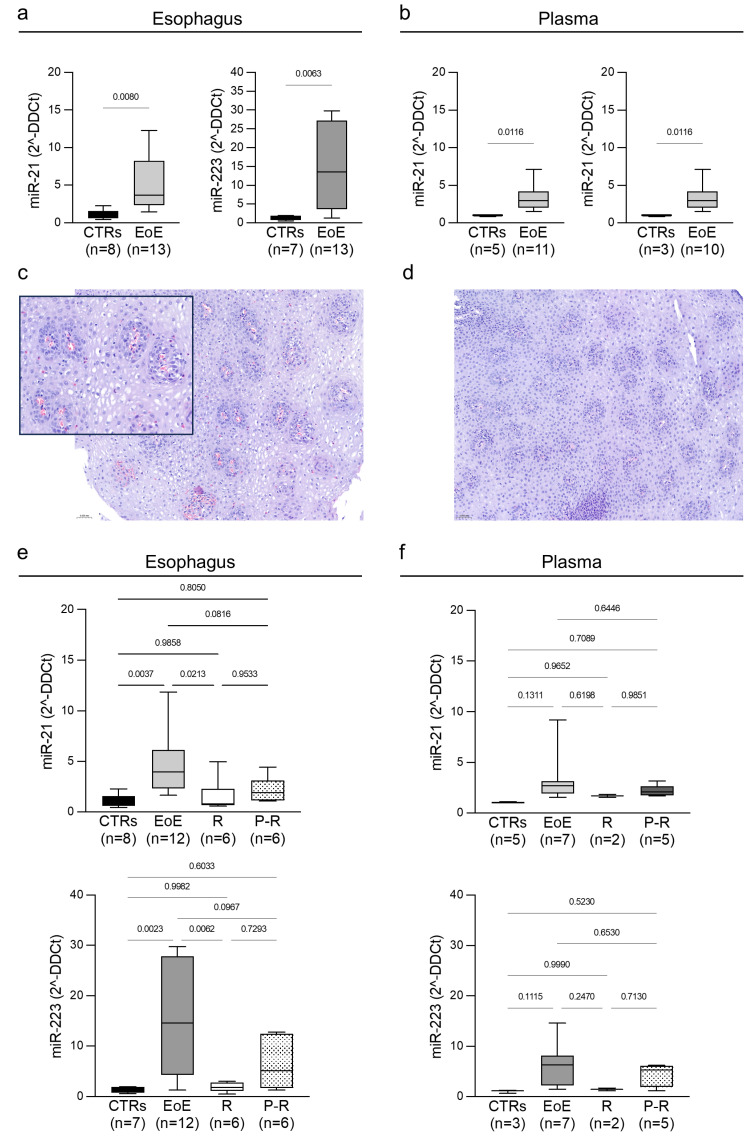
Box plots of microRNAs (miRNA) expression levels in esophageal mucosa (**a**) and in plasma (**b**) from controls samples and Eosinophilic Esophagitis (EoE) patients. (**c**) Fragments of acanthotic and papillomatous squamous epithelium of the esophagus, with a count of intraepithelial eosinophilic granulocytes exceeding the cut-off of 15/HPF, and a maximum concentration of 38/HPF (persistent disease) with a zoomed cropped detail. (**d**) Fragments of squamous epithelium with a preserved histological structure, without intraepithelial eosinophilic granulocytes (remission). Magnification 20×, scale bar 0.050 nm. miR-21-5p and miR-223-3p of treated EoE patients in esophageal mucosa (**e**) and plasma (**f**). Patients showing complete histological remission are referred to as EoE treated R; poor histological responders are indicated as P-R. Statistically significant comparative *p*-values are indicated.

We first compared the expression of miR-21-5p and miR-223-3p in esophageal mucosa and in plasma from EoE subjects and non-EoE controls. In the esophageal mucosa from EoE patients, the expression levels of both miR-21-5p and miR-223-3p were significantly than the non-EoE controls (*p* < 0.005) (Figure 1a). We also observed a significant increase in miR-21-5p and miR-223-3p levels in plasma (Figure 1b). Importantly, tissue and plasma levels exhibited parallel and consistent increases, suggesting that plasma miR-21-5p and miR-223-3p levels reflect those found in esophageal mucosa, and that plasma samples may serve as easily accessible specimens for the monitoring of their tissue levels in EoE patients.

To evaluate possible correlations between esophageal miRNAs and clinical or histological manifestations, we first conducted a Pearson’s correlation analysis that revealed a moderate positive correlation between miR-223-3p expression and the esophageal eosinophil count. To exclude potential confounding factors, we also conducted a multiple linear regression analysis. This allowed us to better isolate the independent effect of the esophageal eosinophil count on miR-223-3p expression while controlling for other variables. According to this analysis, miR-223-3p expression was significantly correlated with peak of the esophagus eosinophil count (Table 2). No correlations were found with clinical symptoms and with signs of atopy.

We also assessed the expression of miR-21-5p and miR-223-3p in the esophageal mucosa and plasma of EoE patients after 8–10 weeks of therapy. Among the participants, four patients received proton pump inhibitors (PPI) (1 mg/kg), seven patients were treated with swallowed fluticasone propionate (220 mcg, two puffs swallowed twice a day), and two patients received both PPI and swallowed fluticasone propionate. Following therapy, 8 of 13 treated patients had a histological remission after therapy, while, in 5 patients, pathological signs of esophagitis persisted (>15 eosinophils/high-power field, HPF). Representative images of hematoxylin/eosin (H/E) staining are shown in Figure 1c,d. Patients with persistent disease showed squamous epithelium of the esophagus with a count of intraepithelial eosinophilic granulocytes exceeding the cut-off of 15/HPF (Figure 1c), while patients in remission showed a preserved histological structure, without intraepithelial eosinophilic granulocytes (Figure 1d).

The esophageal expression of both miR-21-5p and miR-223-3p significantly decreased compared to the levels before treatments (Figure 1e) in patients showing complete histological remission (EoE treated R) and lost statistical significance compared to control values in patients with persistent eosinophil infiltration (EoE treated P-R).

For seven patients, we collected plasma samples before and after treatment. In two of them, we observed complete histological remission, in parallel with the normalization of plasma miR-21-5p and miR-223-3p; meanwhile, in plasma from the five patients with persistent eosinophilic infiltration, miRNA levels remained comparable to the pre-treatment levels (Figure 1f). The combination of these results suggests that plasma levels of these miRNAs reflect changes in tissue levels and may be an additional parameter in the evaluation of patient response to therapies.

**Figure 2:**
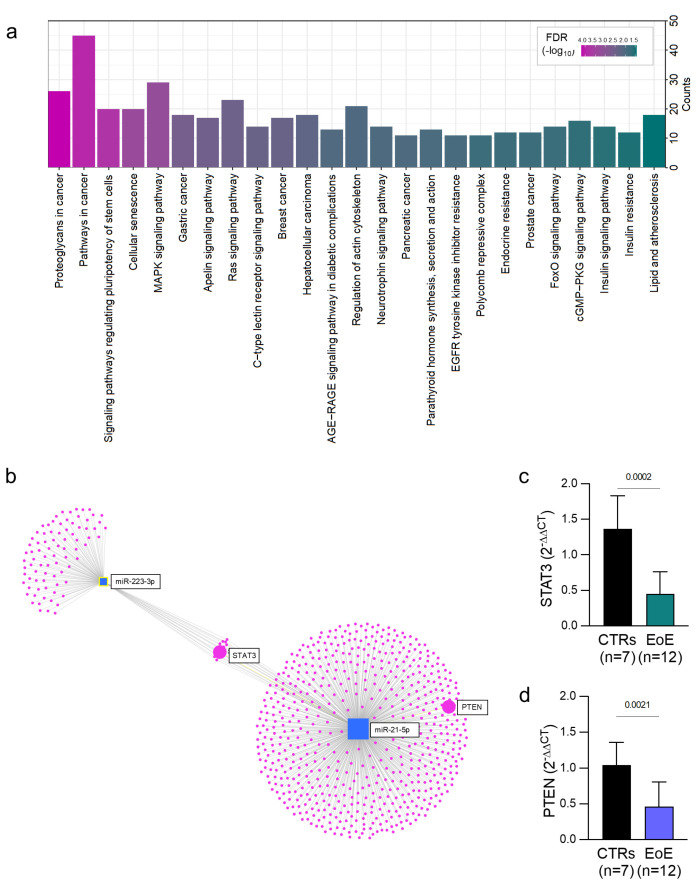
Enrichment analysis of target genes of DE-miRNAs. (**a**) Significant KEGG pathways with FDR < 0.05 are shown; the significance increases change from green to purple. (**b**) miRNA–gene interaction network of miR-21-5p and miR-223-3p. Square shapes represent miRNAs, and circles represent genes regulated by specific miRNAs. The size of the squares depicts the number of miRNA–gene interactions based on the miRTarBase v8.0 data. Expression level of *STAT3 (Signal Transducer and Activator of Transcription 3)*, (**c**) and *PTEN Phosphatase and Tensin Homolog* (**d**) according to real-time qPCR of esophageal tissues from EoE patients and controls. Data are shown as the mean ± SD. Statistically significant comparison *p*-values are indicated.

To further characterize the potential of the two miRNAs as biomarkers, and to analyze their role in pathways that may be relevant for EoE pathophysiology, we conducted an in-depth investigation into their functions and involvement in biological pathways. By conducting a bioinformatic analysis, we identified the predicted target genes of each miRNA (respectively, 384 and 415 target genes of miR-21-5p and miR-223-3p). Subsequently, we identified the most significant pathways in which these genes are implicated. Significant pathways with a false discovery rate (FDR) > 0.05 are shown in Figure 2a. Our analysis revealed that miR-21-5p and miR-223-3p are involved in signaling pathways such as MAPK (Mitogen-Activated Protein Kinase), Ras (Rat Sarcoma), and FoxO (Forkhead Box O), which have potential relevance for the pathophysiology of the condition. For example, the MAPK and FoxO pathways are involved in oxidative stress and in esophageal fibrosis, while the cGMP-PKG or AGE-RAGE pathways are known to be implicated in gastric function and in the pathogenesis of Barrett’s esophagus [15].

Some genes were identified as common targets of miR-21-5p and miR-223-3p, including *PARP1 (Poly [ADP-Ribose] Polymerase 1)*, *RHOB (Ras Homolog Family Member B)*, *E2F1 (E2F Transcription Factor 1)*, *FOXO1 (Forkhead Box O1)*, *FOXO3 (Forkhead Box O3)*, *MKNK2 (MAP Kinase Interacting Serine/Threonine Kinase 2)*, *IGF1R (Insulin-like Growth Factor 1 Receptor)*, *CAPRIN1 (Cell Cycle Associated Protein 1)*, *MEF2C (Myocyte Enhancer Factor 2C)*, *SEPT2 (Septin 2)*, *NFIA (Nuclear Factor I A)*, *ABCB1 (ATP Binding Cassette Subfamily B Member 1)*, *SP1 (Specificity Protein 1)*, *STAT3 (Signal Transducer and Activator of Transcription 3)*, *ASCISBP2L (Apoptosis-Associated Speck-Like Protein Containing a Card Domain 2-Like)*, and *ZNF460 (Zinc Finger Protein 460)* (depicted by dots in Figure 2b). Among these genes, we further analyzed tissue levels of *STAT3*, a protein known to play a role in inflammation, which is activated through phosphorylation in response to various cytokines and growth factors. STAT3 expression was downregulated in the esophageal mucosa of our pediatric EoE cohort compared to controls (Figure 2c). Additionally, the expression levels of *PTEN* (Phosphatase and Tensin Homolog), a known tumor suppressor and regulator of the cell cycle, and a target gene of miR-21-5p, were downregulated in esophageal tissues from EoE patients compared to controls (Figure 2d) [16]. The *PTEN* gene has been implicated in the pathogenesis of different eosinophilic gastroenteropathies, including eosinophilic esophagitis [17], and in the regulation of chemotactic responses of leukocytes through its effect on the Rho GTPase/Rac pathway, which is fundamental for the chemotaxis and activation response of eosinophils [18].

## 3. Discussion

The present study provides insights into the expression of miR-21-5p and miR-223-3p in the esophageal mucosa and in plasma from EoE patients. These miRNAs are implicated in pathways that are expected to be relevant to the disease’s pathophysiology, such as MAPK, Ras, FoxO, oxidative stress, and others.

Although the dysregulation of miR-21-5p and miR-223-3p in adult patients with eosinophilic esophagitis (EoE) is not a novel finding [10,12,14], our study adds to the understanding of their potential as disease biomarkers. Specifically, we demonstrated that, in pediatric EoE patients, plasma levels of miR-21-5p and miR-223-3p mirror those observed in the esophageal mucosa and correlate with patient responses to therapy, as determined by traditional eosinophil counts in esophageal histology. Our results may have clinical implications. The diagnosis and management of EoE typically rely on esophago-gastroduodenoscopy with tissue biopsy. However, these procedures are invasive and have a significant impact on patients’ quality of life, particularly when close and frequent monitoring of the disease status is required. In pediatric patients, the impact of invasive procedures is even heavier, and the availability of markers that are detectable in easily accessible samples, such as blood and plasma, would be most welcome.

Additionally, we evaluated the expression levels of two direct target genes of our upregulated miRNAs: *STAT3*, a target gene of both miR-21-5p and miR-223-3p, and *PTEN*, an miR-21-5p-specific target gene. Although the function of *STAT3* has primarily been implicated in the differentiation of TH17 helper T cells and in the etiopathogenesis of autoimmune diseases [19], recent studies have implicated the *STAT3* signaling pathway in the Th2-mediated response in certain allergic disorders, such as allergic rhinitis [20]. In our analysis, *STAT3* was downregulated in the esophageal mucosa of EoE patients, suggesting an additional or alternative miR-21- and miR-223-dependent modulation of this pathway. A negative relationship between the expression of miR-21-5p and the *PTEN* gene has been observed [21]. In our study, we demonstrated that *PTEN* expression was significantly reduced in patients with EoE compared to controls, consistent with the up-regulation of miR-21-5p, supporting the hypothesis that miR-21-5p may act on PTEN functions.

These findings suggest that miR-21-5p and miR-223-3p act in the relevant pathways and, together with *STAT3* and *PTEN*, may be involved in EoE pathophysiology.

Based on the results of our study, we propose that the quantitative analysis of plasma miR-21-5p and miR-223-3p may serve as a valuable and complementary tool for monitoring disease activity in treated patients. Despite the limitations associated with the small sample size, our study adds further information to previous studies and supports the possibility of obtaining samples from pediatric subjects at the time of EoE diagnosis and after therapies, a noteworthy finding in view of a possible clinical translation. However, studies involving larger patient cohorts are needed to investigate potential correlations between clinical manifestations and plasma miRNA levels, which our study could not establish, and to fully elucidate the role of miRNAs in the disease progression and in EoE patient management.

## 4. Materials and Methods

### 4.1. Study Design, Patients, and Data Collection

We conducted a multicenter prospective observational study. Eligible patients were recruited at the Pediatric Gastroenterology Units of University of Campania “Luigi Vanvitelli” (Naples, Italy) and of University Federico II (Naples, Italy), between January 2022 and January 2023. We enrolled consecutive pediatric subjects with a new diagnosis of active EoE. Inclusion criteria were age  <  18 years and diagnosis of EoE with >15 eosinophils/HPF in the esophageal biopsies, made according to consensus guidelines [16].

The non-EoE controls were subjects with upper gastrointestinal symptoms who had normal endoscopic and histological findings, with a conclusive diagnosis of functional dyspepsia.

At the time of the enrollment, baseline patient characteristics were extracted from medical records: age, sex, disease duration, and history of atopy. Patient demographic and clinical information are provided in Table 1.

Esophageal biopsies and blood samples were obtained during the upper endoscopy, which was performed according to standard clinical protocols for monitoring the patients. Esophageal biopsies were stored in RNA later and frozen at −80 °C until they were processed. Blood samples were centrifuged at 3000 rpm for 10 min to remove blood cells and stored at −80 °C until they were processed.

The study protocol, subject information sheet, informed consent form, and clinical charts were reviewed and approved by the Ethics Committee of the University of Campania “Luigi Vanvitelli” and University “Federico II”, Naples, Italy (Prot. nr. 0008878 del 19 May 2017). The study was performed in accordance with the Helsinki Declaration (2004 Tokyo revision) and with the pertinent European and Italian regulations regarding privacy.

### 4.2. RNA Isolation and Quantitative Real-Time Polymerase Chain Reaction (qRT-PCR)

Total RNA, including small RNAs, was extracted from tissues using the miRNeasy Kit (Qiagen, Germantown, MD, USA) according to the manufacturer′s instructions. For the plasma samples, EDTA was used as an anticoagulant. The expression of mature miRNAs was assayed using a Taqman Advanced MicroRNA Assay (Applied Biosystems, Foster City, CA, USA) specific for miR-21-5p and miR-223-3p (477983_miR, 477975_miR), as described in Tarallo et al. 2019 [6]. In the plasma samples, to calculate ΔCts values, the average of two normalizers was used: a spike in ce-miR-39 and endogenous stable miRNA miR-93. For gene expression, 1 μg of RNA was used to generate cDNA with a QuantiTect Reverse transcription Kit (Qiagen, Hilden, NRW, Germany) and analyzed via qRT-PCR using SYBR Green master mix (Roche, Basel, Switzerland). All data were normalized to *GAPDH*. Primers are listed below: *STAT3* (Fw: TGGCCCAATGGAATC; Rv: ACTGCTGGTCAATCT), *PTEN* (Fw: ACGACGGGAAGACAA; Rv: AGGTTTCCTCTGGTCCTGGT), *GAPDH* (Fw: GGGCCAGGTCATCCCTGA; Rv: GCCTGCTTCACCACCTTC). Differences in miRNAs and gene expression, expressed as fold changes, were calculated using the 2^−ΔΔCt^ method.

### 4.3. Bioinformatic Analysis

KEGG (Kyoto Encyclopedia of Genes and Genomes) pathway enrichment analysis of the miRNA targets was performed using David (Database for Annotation, Visualization, and Integrated Discovery) bioinformatics, TargetScan with FDR < 0.05 as threshold for significant enrichment, and the SR-PLOT tool for graphical representation. TO visualize and identify the interactions of the miR-21-5p and miR-223-3p target genes, we used miRNet tools (network-based visual analytics for microRNA functional analysis and systems biology) [22].

### 4.4. Histological Analysis

Hematoxylin/eosin (H&E) staining (Sigma-Aldrich, St. Louis, MO, USA) was carried out in one distal esophageal biopsy per patient according to standard procedures [23].

### 4.5. Statistical Analysis

Statistical analysis was performed using GraphPad Prism version 10.4.(GraphPad Software, LLC, San Diego, CA, USA) Student’s *t* test or group-wise comparisons by two-way analysis of variance (ANOVA) with Tukey post hoc test were performed for the data shown in Figure 1. A multiple linear regression analysis was calculated in R version 4.3.2 (Foundation for Statistical Computing, Vienna, Austria).

## Figures and Tables

**Table 1:** Characteristics of EoE patients.

	EOE Patients *n* = 13
**Sex males, *n* (%)**	8 (61)
**Age years, median ± SD**	13 ± 4.9
**History of atopy, n (%)**	9 (69)
- Asthma	3 (27.1)
- Allergic rhinitis	5 (38.4)
- Atopic dermatitis	3 (27.1)
**Comorbidities (%)**	5 (38.5)
- Celiac disease	3 (23)
- Diabetes Mellitus Type 1	4 (30.8)
**EoE treatment, *n* (%)**	
- PPI	4 (30.7)
- Topical CCS	7 (53.8)
- PPI + topical CCS	2 (15.3)
**Eos/HPF (mean)**	
T0 biopsy	33.75
T1 biopsy	13.23
**EREFS (mean)**	
T0	2.07
T1	1.53

EREFS: Endoscopic reference score.

**Table 2:** Multiple linear regression model.

Esophageal miR-223-3p	Estimate	*p* Value
(Intercept)	−24.595264	0.2176
EC-T0_Blood	0.011237	0.2752
EC-T0_Esophagus	1.015288	0.0318 *
Allergy	6.610019	0.4009
EREFS-T0	−8.176028	0.07
CD	11.834543	0.272
DM1	7.98814	0.3675

(*) denotes statistical significance (*p* < 0.05). EC = Eosinophilic Count; CD = Celiac Disease; DM = Diabetes Mellitus type 1; EREFS: Endoscopic reference score.

## Data Availability

The data sets generated during and/or analyzed in the current study are available from the corresponding author upon reasonable request.

## Abbreviations

The following abbreviations are used in this manuscript:

CCS	corticosteroids
EoE	eosinophilic esophagitis
FDR	false discovery rate
H/E	hematoxylin/eosin
HPF	high-power field
miRNA	MicroRNA
PPI	proton pump inhibitors
qRT-PCR	quantitative real-time polymerase chain reaction
KEGG	Kyoto Encyclopedia of Genes and Genomes
David	Database for Annotation, Visualization, and Integrated Discovery

## References

[1] Furuta G.T., Katzka D.A. (2015). Eosinophilic Esophagitis. N. Engl. J. Med..

[2] Spergel J.M., Brown-Whitehorn T.A., Muir A., Liacouras C.A. (2020). Medical algorithm: Diagnosis and treatment of eosinophilic esophagitis in children. Allergy.

[3] Dellon E.S., Hirano I. (2018). Epidemiology and Natural History of Eosinophilic Esophagitis. Gastroenterology.

[4] Gupta S.K. (2008). Noninvasive markers of eosinophilic esophagitis. Gastrointest. Endosc. Clin. N. Am..

[5] Ho P.T.B., Clark I.M., Le L.T.T. (2022). MicroRNA-Based Diagnosis and Therapy. Int. J. Mol. Sci..

[6] Tarallo A., Carissimo A., Gatto F., Nusco E., Toscano A., Musumeci O., Coletta M., Karali M., Acampora E., Damiano C. (2019). microRNAs as biomarkers in Pompe disease. Genet. Med..

[7] Bartel D.P. (2004). MicroRNAs: Genomics, biogenesis, mechanism, and function. Cell.

[8] Allegra A., Alonci A., Campo S., Penna G., Petrungaro A., Gerace D., Musolino C. (2012). Circulating microRNAs: New biomarkers in diagnosis, prognosis and treatment of cancer (review). Int. J. Oncol..

[9] Markey G.E., Donohoe C.L., McNamee E.N., Masterson J.C. (2023). MicroRNA dysregulation and therapeutic opportunities in esophageal diseases. Am. J. Physiol. Gastrointest. Liver Physiol..

[10] Jhaveri P.B., Lambert K.A., Bogale K., Lehman E., Alexander C., Ishmael F., Jhaveri P.N., Hicks S.D. (2023). Salivary microRNAs in pediatric eosinophilic esophagitis. Allergy Asthma Proc..

[11] Votto M., Strisciuglio C., Indolfi C., Marseglia G.L., Miraglia Del Giudice M., Licari A. (2024). Correspondence to “Salivary Immunoinflammatory Proteins Identify Children with Eosinophilic Esophagitis”. Allergy.

[12] Zahm A.M., Menard-Katcher C., Benitez A.J., Tsoucas D.M., Le Guen C.L., Hand N.J., Friedman J.R. (2014). Pediatric eosinophilic esophagitis is associated with changes in esophageal microRNAs. Am. J. Physiol. Gastrointest. Liver Physiol..

[13] Cañas J.A., Tabares A., Barbero C., García-Sánchez D., Sastre B., Rodrigo-Muñoz J.M., Mahíllo-Fernández I., Rayo A., Borrell B., Cilleruelo M.L. (2020). Proton-pump Inhibitor Response Prediction Using Esophageal microRNAs in Children With Eosinophilic Esophagitis. J. Pediatr. Gastroenterol. Nutr..

[14] Lu T.X., Sherrill J.D., Wen T., Plassard A.J., Besse J.A., Abonia J.P., Franciosi J.P., Putnam P.E., Eby M., Martin L.J. (2012). MicroRNA signature in patients with eosinophilic esophagitis, reversibility with glucocorticoids, and assessment as disease biomarkers. J. Allergy Clin. Immunol..

[15] Zou K., Dong H., Li M., Zhang Y., Zhang K., Song D., Chu C. (2023). Comprehensive analysis of transcriptome-wide N6-methyladenosine methylomes in the Barrett’s esophagus in rats. Genomics.

[16] Dellon E.S., Liacouras C.A., Molina-Infante J., Furuta G.T., Spergel J.M., Zevit N., Spechler S.J., Attwood S.E., Straumann A., Aceves S.S. (2018). Updated International Consensus Diagnostic Criteria for Eosinophilic Esophagitis: Proceedings of the AGREE Conference. Gastroenterology.

[17] Wu Y., Song Y., Xiong Y., Wang X., Xu K., Han B., Bai Y., Li L., Zhang Y., Zhou L. (2017). MicroRNA-21 (Mir-21) Promotes Cell Growth and Invasion by Repressing Tumor Suppressor PTEN in Colorectal Cancer. Cell. Physiol. Biochem..

[18] Li Z., Dong X., Wang Z., Liu W., Deng N., Ding Y., Tang L., Hla T., Zeng R., Li L. (2005). Regulation of PTEN by Rho small GTPases. Nat. Cell Biol..

[19] Yang X.O., Panopoulos A.D., Nurieva R., Chang S.H., Wang D., Watowich S.S., Dong C. (2007). STAT3 regulates cytokine-mediated generation of inflammatory helper T cells. J. Biol. Chem..

[20] Wang J., Shen Y., Li C., Liu C., Wang Z.H., Li Y.S., Ke X., Hu G.H. (2019). IL-37 attenuates allergic process via STAT6/STAT3 pathways in murine allergic rhinitis. Int. Immunopharmacol..

[21] Meng F., Henson R., Wehbe-Janek H., Ghoshal K., Jacob S.T., Patel T. (2007). MicroRNA-21 regulates expression of the PTEN tumor suppressor gene in human hepatocellular cancer. Gastroenterology.

[22] Chang L., Zhou G., Soufan O., Xia J. (2020). miRNet 2.0: Network-based visual analytics for miRNA functional analysis and systems biology. Nucleic Acids Res..

[23] Hasan S.H., Taylor S., Garg S., Buras M.R., Doyle A.D., Bauer C.S., Wright B.L., Schroeder S. (2021). Diagnosis of Pediatric Non-Esophageal Eosinophilic Gastrointestinal Disorders by Eosinophil Peroxidase Immunohistochemistry. Pediatr. Dev. Pathol..

